# A Putative C_2_H_2_ Transcription Factor CgTF6, Controlled by CgTF1, Negatively Regulates Chaetoglobosin A Biosynthesis in *Chaetomium globosum*

**DOI:** 10.3389/ffunb.2021.756104

**Published:** 2021-10-15

**Authors:** Yu Yan, Biyun Xiang, Qiaohong Xie, Yamin Lin, Guangya Shen, Xiaoran Hao, Xudong Zhu

**Affiliations:** ^1^Beijing Key Laboratory of Genetic Engineering Drug and Biotechnology, College of Life Sciences, Beijing Normal University, Beijing, China; ^2^Xiamen No. 1 High School of Fujian, Xiamen, China; ^3^Shenzhen Senior High School Group, Shenzhen, China; ^4^National Experimental Teaching Demonstrating Center, College of Life Sciences, Beijing Normal University, Beijing, China

**Keywords:** chaetoglobosin A, transcription factor, C_2_H_2_, Zn(II)_2_Cys_6_, *Chaetomium globosum*, secondary metabolites, sporulation

## Abstract

Gα signaling pathway as well as the global regulator LaeA were demonstrated to positively regulate the biosynthesis of chaetoglobosin A (ChA), a promising biotic pesticide produced by *Chaetomium globosum*. Recently, the regulatory function of Zn_2_Cys_6_ binuclear finger transcription factor *CgcheR* that lies within the ChA biosynthesis gene cluster has been confirmed. However, *CgcheR* was not merely a pathway specific regulator. In this study, we showed that the homologs gene of *CgcheR* (designated as *Cgtf1*) regulate ChA biosynthesis and sporulation in *C. globosum* NK102. More importantly, RNA-seq profiling demonstrated that 1,388 genes were significant differentially expressed as *Cgtf1* deleted. Among them, a putative C_2_H_2_ transcription factor, named *Cgtf6*, showed the highest gene expression variation in zinc-binding proteins encoding genes as *Cgtf1* deleted. qRT-PCR analysis confirmed that expression of *Cgtf6* was significantly reduced in CgTF1 null mutants. Whereas, deletion of *Cgtf6* resulted in the transcriptional activation and consequent increase in the expression of ChA biosynthesis gene cluster and ChA production in *C. globosum*. These data suggested that CgTF6 probably acted as an end product feedback effector, and interacted with CgTF1 to maintain a tolerable concentration of ChA for cell survival.

## Introduction

*Chaetomium globosum* is ubiquitous in the environment (Wang X. W. et al., 2016). With a large number of structurally diverse metabolites, *C. globosum* has attracted substantial attention for its great potential in the biocontrol of nematode, sap-sucking pests and phytopathogenic fungi (Qi et al., 2011; Hu et al., 2012; Uzma et al., 2018). For example, the culture filtrates of *C. globosum* is demonstrated to strongly inhibit the egg hatching of root-knot nematode *Meloidogyne incognita* and soybean cyst nematode *Heterodera glycines* (Meyer et al., 2004). In our laboratory, the culture filtrates of *C. globosum* NK102, an endophyte formally isolated as a high efficiency lignocellulose degrading fungus, exhibited strong nematicidal activity against *M. incognita*, and the main active component was identified as chaetoglobosin A [ChA, the median lethal concentration (LC_50_) is 77.0 μg/ml] (Hu et al., 2013, 2018). ChA belongs to the family of cytochalasans, which are known to interact with the actin filament network by capping their growing ends, and thereby blocking the assembly or disassembly of the microfilaments resulting in altered dynamic properties (Scherlach et al., 2010). Like most cytochalasans, ChA has been confirmed to target filamentous actin in mammalian cells, and thereby induces cell-cycle arrest and inhibits membrane ruffling and cell migration (Knudsen et al., 2014), endowing ChA strong cytotoxicity against tumor cell lines, immunomodulatory activities, and antifungal activities (Sekita et al., 1982; Jiao et al., 2004; Huang et al., 2016). Not only ChA, other secondary metabolites of *C. globosum*, e.g., chaetoglobosin B and flavipin are also exhibited strong nematicidal activities against the second stage juveniles of *M. javanica*, with LC_50_ values of 107.7 and 99.2 μg/ml after 72 h, respectively (Khan et al., 2019). The nematophagous and entomogenous fungi can produce secondary metabolites to infect or kill nematodes, and some of these fungi have been or are being developed as biological control agents in China and worldwide (Sharon et al., 2001; Degenkolb and Vilcinskas, 2016). From the fermentation broth of the destructive parasitic fungus of the cereal cyst nematode *H. filipjevi*, Ashrafi extracted ChA, and demonstrated ChA caused a temporary immobilization on the second stage juveniles (Ashrafi et al., 2017). Taken together, published data clearly demonstrate that it is possible to explore the nematicidal activity of *C. globosum*, and ChA can be used as a biological agent for the control of plant pathogenic microorganisms and pests.

**Figure 1:**
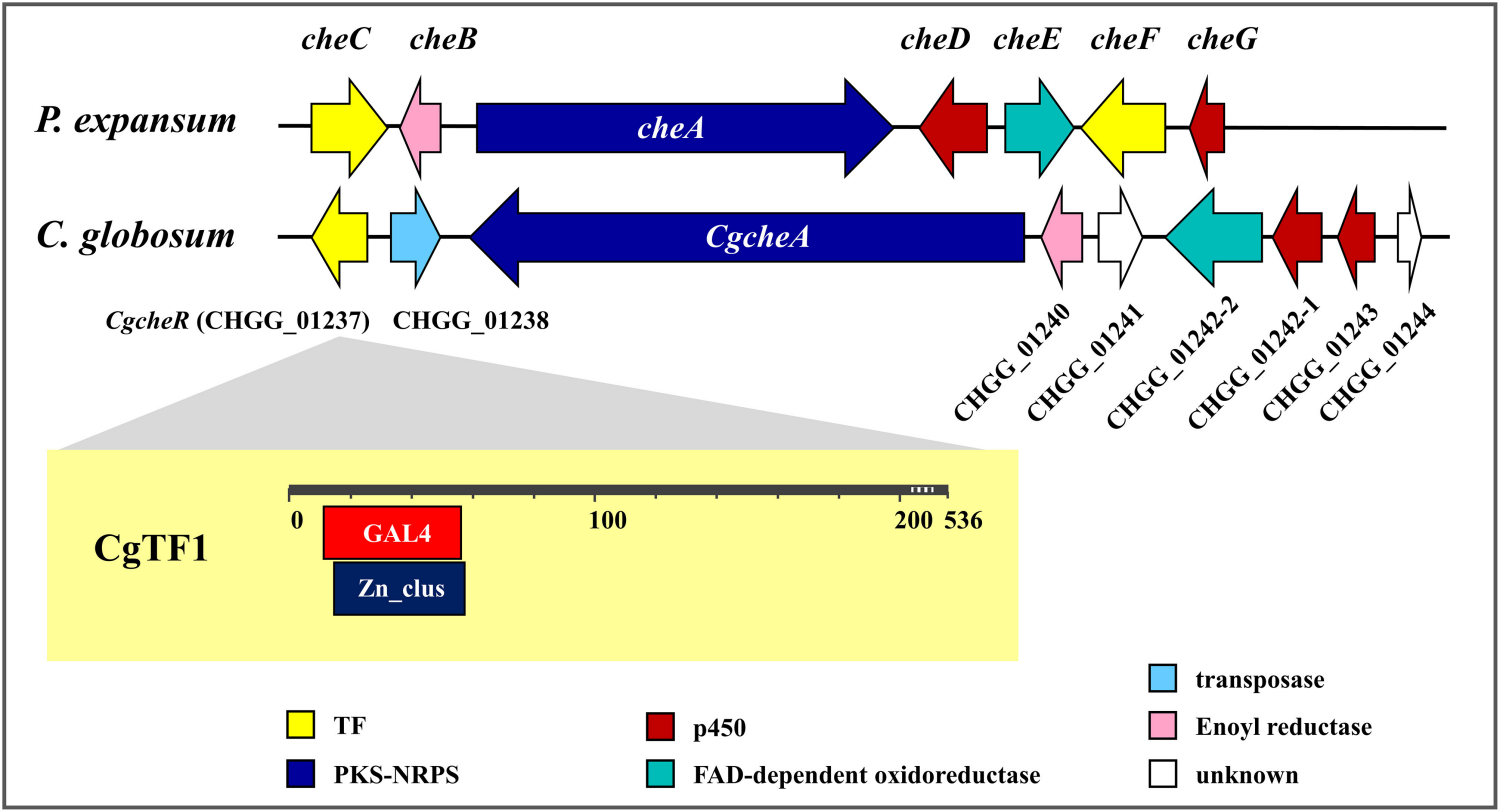
Chaetoglobosin A biosynthetic gene clusters in *Penicillium expansum* and *Chaetomium globosum*.

The genetic and molecular basis for ChA biosynthesis has been revealed (Figure 1). Like other members of this group, ChA is structurally a fungal alkaloid with an isoindole moiety fused to a macrocycle. The gene cluster responsible for ChA biosynthesis was predicted and identified using a siRNA technology in *Penicillium expansum* (Schümann and Hertweck, 2007). The carbon scaffold of ChA is synthesized by a hybrid iterative type I polyketide synthase-non-ribosomal peptide synthetase (PKS-NRPS) CheA and a stand-alone enoyl reductase CheB. After a spontaneous intramolecular condensation and a Diels-Alder reaction, the key intermediate prochaetoglobosin generated, which can be furnished into ChA after a set of oxidative modifications. Recently, gene disruption studies of *C. globosum* led to a more sophisticated biosynthetic insight of ChA. Homologs of *CheA* and *CheB* as well as the enzymes involved in the final oxidative transformations are identified and characterized in *C. globosum* (Ishiuchi et al., 2013). Nonetheless, we previously observed in *C. globosum* NK102 that a conidial pigment polyketide synthase gene, *pks-1/alb1*, was also required for ChA biosynthesis (Hu et al., 2012). Knock down of *pks-1* resulted in dramatically reduction of ChA production and significant inhibition of pigmentation and sporulation. In filamentous fungi, genes within a biosynthetic gene cluster are often co-regulated by epigenetic modification, signal transduction and transcription factors. The interaction of these factors usually determines the type and amount of mycotoxins produced by a single strain (Keller, 2019). Targeted gene deletion in *C. globosum* demonstrated a few proteins probably involved in the regulation of ChA biosynthesis (Nakazawa et al., 2013). For example, the light-regulated developmental factor CgVeA, and the global regulator of secondary metabolites CgLaeA, two main components of the conserved global regulatory unit known as the *velvet* complex, are demonstrated to be required for ChA biosynthesis. Deletion of *CgveA* led to loss of production of ChA as well as three other metabolites. A more significant effect was observed in *CglaeA* deletants, which were aberrant in secondary metabolism and lost the ability of producing metabolites, including ChA. A complete inhibition of ChA cluster gene expression was also observed in *CgveA* or *CglaeA* deletants. Overexpression of *CglaeA* conducted by Jiang T. et al. (2016) provided more evidence that *CglaeA* acted as a global regulator controlling secondary metabolites biosynthesis in *C*. *globosum*. In addition, a histone acetyltransferase encoded by *CgsptJ* has also been shown to be necessary for ChA biosynthesis. Deletion of *CgsptJ* resulted in inhibition of expression of CHGG_01241, CHGG_01242 and CHGG_01243, and aberrant in ChA production. Recently, we reported the heterotrimeric Gα-cAMP/PKA signaling pathway positively regulated pigmentation, ChA production and sexual development in *C. globosum* (Hu et al., 2018). Knockdown of a Gα-encoding gene *gna-1* led to decreased production of ChA and significantly fall in expression of corresponding genes, e.g., *pks-1* and *CgcheA*. These defects could be restored either by simultaneous knockdown of the *pkaR* gene encoding a regulatory subunit of cAMP-dependent protein kinase A (PKA) or be supplemented with a cAMP analog, 8-Br-cAMP. Moreover, expression of *CglaeA* was down regulated in *gna-1* silent mutants and restored to wild-type level when *pkaR* was silenced simultaneously. Knockdown of *gna-1* also led to decreased expression of *CgveA* and *CgsptJ*. These results suggesting that CgLaeA, CgVeA and CgSptJ probably work as downstream effectors that dictate expression of the ChA biosynthesis gene cluster by interacting with Gα-cAMP/PKA signaling.

Transcription factors (TFs) also play an important role in the regulation of secondary metabolites biosynthesis. TFs are proteins that binds to specific DNA sequences, thereby controlling the flow of genetic information from DNA to mRNA (Lu et al., 2014). Approximately, up to 50% of fungal biosynthetic gene clusters contain a cluster-specific transcription factor (Keller, 2019). Zinc-binding proteins are one of the largest families of transcriptional regulators in eukaryotes. Of the DNA (or RNA)-binding variety, three major classes of zinc finger proteins have been established, based on their unique and highly conserved consensus amino acid sequences. Class I encompasses the Cys_2_His_2_ (C_2_H_2_) proteins and is often referred to as the classical zinc finger. Class II represents the Cys_4_ (C_4_) zinc fingers. Class III (C_6_) zinc finger proteins, also known as zinc binuclear cluster, or Zn(II)_2_Cys_6_ (Zn_2_C_6_) proteins, contain a DNA-binding domain (DBD) that consists of six cysteine residues bound to two zinc atoms. They are strictly fungal proteins (MacPherson et al., 2006). The *Saccharomyces cerevisiae* transcription factor Gal4p is arguably the most well-known and well-studied zinc cluster protein, mainly targets and activates the transcription of *GAL1* (encoding galactokinase), *GAL7* (galactose-1-phosphate uridyltransferase), and *GAL10* (UDP-glucose 4-epimerase) to regulate the metabolism of galactose. In filamentous fungi, TFs are frequently documented as regulators of secondary metabolites biosynthesis or drug resistance, e. g., AflR and AtrR, two well-known Zn_2_Cys_6_ transcription factors in *Aspergillus spp*. AflR activates expression of sterigmatocystin biosynthesis genes while its transcription is regulated by LaeA (Shwab and Keller, 2008). On the other hand, AflR negatively regulates LaeA expression in a unique feedback loop (Bok and Keller, 2004). AtrR has been reported to be involved in drug resistance. Deletion of the *atrR* gene in *A. oryzae, A. nidulans* and *A. fumigatus* resulted in hypersensitivity to antifungal drugs (Hagiwara et al., 2017).

Two putative TFs (CheC and CheF) encoding gene are located within the ChA biosynthetic gene cluster in *P. expansum*, while a single putative TF CHGG_01237 lies in the ChA biosynthetic gene cluster in *C. globosum* (Figure 1). Recently, regulatory function of CHGG_01237 (designated as *CgcheR*) on ChA biosynthesis has been confirmed (Cheng et al., 2021). It was demonstrated that CgCheR activates the transcription of ChA biosynthetic genes in a pathway-specific manner. Here, a similar work has been done on *Cgtf1*, a homologous gene of *CgcheR* in *C. globosum* NK102. The regulatory role of *Cgtf1* on ChA biosynthesis was confirmed by detailed phenotypic characterization of its deletion mutants. However, CgTF1 was not merely a pathway specific regulator. A total of 1,388 genes were significant differentially expressed as CgTF1 deleted. More importantly, transcription of a putative Cys_2_His_2_ transcription factor CgTF6 (gene locus: CHGG_07161) was found to be downregulated in CgTF1 null mutant according to RNA-Seq Profiling and qRT-PCR. Subsequent targeted gene deletion of *Cgtf6* was conducted. According to qRT-PCR analysis and characterization of the ChA production as well as associated genes expression responsive to *Cgtf6* deletion, a possible mechanism was proposed to explain CgTF1 and CgTF6 action in regulation of ChA biosynthesis.

## Materials and Methods

### Strains and Culture Conditions

The wild-type strain *C. globosum* NK102, isolated and stocked by our laboratory was used as the host strain for gene deletion experiments in this study. NK102 was grown on potato dextrose agar (PDA) at 28°C. For preparation of protoplasts, approximately (1~4) × 10^4^ ascospores, collected from a single plate were used to inoculate 100 ml of potato dextrose broth (PDB) medium, shaken at 28°C and 200 rpm, for 2 days. Ten grown mycelial balls were transferred into 100 ml fresh PDB medium, continued to grow for 3 days at 28°C with a shaking speed of 160 rpm. For DNA isolation, 5-mm agar plaques containing the fungal hypha were inoculated in 100 ml PDB and incubated for 4 days in a rotary shaker at 28°C and 200 rpm. Under identical culture conditions, mycelium from 4 days cultures were chosen to isolate RNA for RNA-seq profiling, and mycelium from 7 days cultures were chosen for qRT-PCR analysis. For high-performance liquid chromatography (HPLC) analysis, strains were cultured in 100 ml PDB for 7 days with shaking at 28°C and 200 rpm. *Escherichia coli*, used for constructing plasmids and conjugation, was grown in Luria-Bertani (LB) broth (Difco) or on LB agar plates at 37°C.

### Plasmids Construction for Gene Deletion

Recently, we successfully established a “suicide” CRISPR-Cas9 system to promote gene deletion in *C. globosum* NK102 (Wang Y. et al., 2016; Xiang et al., 2021). Plasmids for *Cgtf1* or *Cgtf6* deletion were constructed based on plasmid pCRISPR-Hyg (Supplementary Figure 1). To generate the *Cgtf1*-targeted gene knockout vector, vector pCRISPR-Hyg was linearized by restriction enzyme *Bbs* I. The GN_19_NGG sequence of *Cgtf1* was searched for designing the complementary primers N19-F and N19-R. The cohesive terminus of *Bbs* I was added at the 5′ and 3′ terminal of each primer, respectively. These primers were synthesized, denatured (95°C, 10 min) and anneal naturally (room time) to generate the GN_19_NGG fragment. The GN_19_NGG fragment was ligated into the *Bbs* I site of pCRISPR-Hyg to generate plasmid PCgtf1-N19. The upstream (753 bp) and downstream (736 bp) flanking sequences of the *Cgtf1* were amplified from NK102 genomic DNA with primers 01237UP-F/01237UP-R and 01237DOWN-F/01237DOWN-R, respectively. PCgtf1-N19 was cut by *Xho* I to generate a large fragment containing Cas9 and a small fragment containing a *Hyg*^*R*^ cassette bearing the hygromycin B resistance marker. The upstream of *Cgtf1, Hyg*^*R*^ cassette, downstream of *Cgtf1* were fused with the large fragment via In-Fusion HD Cloning kits (Clontech, California, USA). The merged plasmid, designated as pCgtf1, was linearized with *Nru* I and introduced into the NK102 via PEG mediated protoplast transformation.

To generate the *Cgtf6*-targeted gene knockout vector, the GN_19_NGG sequence of *Cgtf6* was searched for designing the complementary primers 07161-N19-F and 07161-N19-R. These primers were synthesized, denatured and anneal naturally to generate the GN_19_NGG fragment. The GN_19_NGG fragment was ligated into the *Bbs* I site of pCRISPR-Hyg to generate plasmid PCgtf6-N19. Nucleotide fragments corresponding upstream (631 bp) and downstream (736 bp) sequences based on the *Cgtf6* sequence were cloned by PCR using *C. globosum* NK102 genomic DNA and pair primers 07161-UP-F/07161-UP-R and 07161-DOWN-F/07161-DOWN-R, respectively. Then the upstream of *Cgtf6, Hyg*^*R*^ cassette, downstream of *Cgtf6* were fused as mentioned above. The merged plasmid, designated as pCgtf6, was linearized with *Nru* I and introduced into the NK102 via PEG mediated protoplast transformation. All primers were listed in Supplementary Table 1.

### Fungal Transformation, Transformants Screening and Identification

Protoplast preparation and transformation were performed as previously described (Hu et al., 2018). Transformants were selected on PDA plates containing 100 μg/ml hygromycin B (Sigma-Aldrich, St Louis, MO, USA).

For screening of the Δ*cgtf1* mutants, three PCR reactions per transformant were carried out, primers T01237up-F/THyg-R and THyg-F/T0127down-R were used to detect homologous recombination of the 5′ and 3′ flanks, respectively, and primers L01237*Kpn* I-F/L01237*Hind* III-R were used to detect the ORF of gene *Cgtf1*. For further confirmation, genomic DNA was extracted, digested and subjected to Southern blot analysis as previously described (Hu et al., 2018). A 695 bp 3′-flank of *Cgtf1* and a 2,124 bp *Hyg*^*R*^ fragment were labeled as probes.

For screening of the Δ*cgtf6* mutants, three PCR reactions per transformant were carried out, primers Cgtf6-Up-Hyg-F/Cgtf6-Up-Hyg-R and Cgtf6-Hyg-Down-F/Cgtf6-Hyg-Down-R were used to detect homologous recombination of the 5′ and 3′ flanks, respectively, and primers qCHGG_07161-F/qCHGG_07161-R were used to detect the ORF of gene *Cgtf6*. For further confirmation, genomic DNA was extracted and subjected to Southern blot analysis as previously described (Hu et al., 2018). Plasmid pCgtf6 was cut by *Xho* I and generated a 2,131 bp *Hyg*^*R*^ cassette, which was subsequently labeled as the probe. Experiments involving DNA labeling, hybridization and detection were carried out according to the instructions of the DIG High Prime DNA Labeling and Detection Starter Kit II (Roche China, Shanghai, China). All primers were listed in Supplementary Table 1.

### RNA Isolation and Quantitative Real-Time (qRT)-PCR

Total RNA was extracted from the lyophilized and ground mycelium using an RNAiso Plus kit (TaKaRa, Beijing, China). The first-strand cDNA was generated by reverse transcription in a 20 μl reaction using Transcript First-Strand cDNA Synthesis Super Mix kit (TransGen Biotech, Beijing, China). Quantitative real-time PCR was performed by Light Cycler^®^ 480 instrument II (Roche China, Shanghai, China). Each reaction of 20 μl PCR was performed with SYBR Green I PCR master mix (Roche China, Shanghai, China). Reactions were set up in three replicates per sample. Controls without addition of the templates were included for each primer set. PCR cycling parameters were: pre-incubation at 94°C for 10 min, followed by 40 cycles of denaturation at 95°C for 10 s, annealing at 55°C for 20 s and extension at 72°C for 20 s. The qRT-PCR data were analyzed using the 2^−ΔΔCt^ relative quantification method (Livak and Schmittgen, 2001) to calculate relative expression levels of genes. The housekeeping genes encoding actin or glyceraldehyde-3-phosphate dehydrogenase (GAPDH) were served as reference. The amplification efficiencies of the target and reference genes were compared at different template concentrations. The gene-specific pairs of primers used in the amplifications were: q01237-F/q01237-R for *Cgtf1*, qCHGG_07161-F/qCHGG_07161-R for *Cgtf6*, qCHGG_01239(s)/qCHGG_01239(as) for *CgcheA*, qPKS(s)/qPKS(as) for *pks-1*, qCHGG_01240-F/qCHGG_01240-R for CHGG_01240, qCHGG_01242-F/qCHGG_01242-R for CHGG_01242, qCHGG_01243-F/qCHGG_01243-R for CHGG_01243, qCHGG_01244-F/qCHGG_01244-R for CHGG_01244, qCHGG_02034-F/qCHGG_02034-R for CHGG_02034, GAPDH-up/GAPDH-down for *GAPDH*, and qActin(s)/qActin(as) for the *ACTIN* gene (Supplementary Table 1).

### Detection of Chaetoglobosin a by High Performance Liquid Chromatography (HPLC)

Each sample of liquid culture was extracted with an equal volume of ethyl acetate. The organic phase was then concentrated, dissolved and centrifuged as previously reported (Hu et al., 2018). The supernatant was filtered through a 0.45 μm Millipore filter and subjected to HPLC analysis on an Agilent 1,200 HPLC system (Agilent Technologies, CA, USA) with a Kromasil C18 ODS column (4.6 × 250 mm, AKZO Nobel, Gland, Switzerland). The UV detection wavelength was set at 227 nm, and the sample flow rate was set at 1 ml/min. Standard ChA (Sigma, St. Louis, USA) served as control. For quantification of ChA, a standard curve was created with known concentrations of the standard sample.

### Quantification of Ascospores Production

Ascospores were harvested from 30-day old cultures on PDA at 28°C in triplicate for each strain. Firstly, 2 ml sterile distilled water was added to each plate. Then, ascospores formed on each plate were scrapped using a sterilized spatula, resuspended in 30 ml sterile distilled water and vortex by a Vortex-Genie 2 Mixer (Scientific Industries, New York, USA) for 1 min at the highest speed. After centrifugation at 3,000 × *g* for 10 min, the supernatant full of hyphae-debris was discarded, and the ascospores pellet was washed twice with sterile distilled water and finally resuspended in 10 ml sterile distilled water. The concentration of ascospores suspension in sterile distilled water was determined by hemocytometry under the Moticam×3 microscope (Motic, Xiamen, China).

### RNA-Seq, Data Mining and Gene Ontology Analysis

RNA-seq profiling was carried out by a commercial provider to monitor the consequences of the *Cgtf1* knock-out. Illumina HiSeq™ sequencing of total mRNA from the wild-type or deficient mutants Δ*cgtf1-*10 and Δ*cgtf1-*14 was conducted by BGI (Shenzhen, China; http://en.genomics.cn/navigation/index.action). Raw reads were counted using SOAPnuke version 1.4.0 (BGI, Shenzhen, China) and then were trimmed using trimmomatic (version 0.36). The resulted clean reads were mapped to the *C*. *globosum* reference genome (CBS 148.51) using HISAT(Hierarchical Indexing for Spliced Alignment of Transcripts), (version 2.1.0) and were aligned using Bowtie 2 (version 2.2.5). Expression values were calculated as fragments per kilo base of transcript per million mapped reads (FPKM) using RSEM (version 1.2.8), (Li and Dewey, 2011). *P*-values were used to evaluate expression differences at a statistically significant level (Mortazavi et al., 2008). A false discovery rate (FDR)-corrected *p*-value ≤ 0.001 and |Log2 (Ratio value)| ≥1 were used to identify the differentially expressed genes (DEGs) and differentially expression tags (DETs).

## Results

### Construction of CgTF1 Deletants via the “Suicide” CRISPR-Cas9 System

The 1611-bp *Cgtf1* gene was cloned, subjected to sequencing and submitted to the GenBank database under accession number MT050051. The predicted protein CgTF1 encoded by *Cgtf1* is a 536 amino acids sequence comprises two overlapping domains, a GAL4-like Zn(II)_2_/Cys_6_ binuclear cluster DNA-binding domain (GAL4, cd00067) and a fungal Zn(II)_2_Cys_6_ binuclear cluster domain (Zn_clus, pfam00172), (Figure 1). The amino acid sequence of CgTF1 is identical to CgCheR. Blastp analysis revealed that CgTF1 had 77.19, 38.10, 30.56, 26.10 and 24.64% identity to a GAL4-like Zn_2_Cys_6_ binuclear cluster DNA-binding domain protein (*C. globosum* E-C-2, QHD43140.1), CheC (*P. expansum*, CAO91859.1), CheF (*P. expansum*, CAO91864.1), GAL4 (*Aspergillus sclerotialis*, RJE27110.1) and Gal4p (*S. cerevisiae*, NP_015076.1), respectively. To characterize the functional roles of CgTF1 on ChA biosynthesis, CgTF1 was deleted using an established “suicide” CRISPR-Cas9 system (Xiang et al., 2021). All gene-targeted plasmids were constructed based on plasmid pCRISPR-Hyg (Supplementary Figure 1), which contain the Cas9 and gDNA expression cassettes and a hygromycin B resistance marker. To target *Cgtf1*, the GN_19_NGG fragment was ligated into pCRISPR-Hyg to generate plasmid PCgtf1-N19, two flanking sequences of the *Cgtf1* were then fused with the hygromycin-resistant gene, respectively. The resulted construct, designated pCgtf1 (Supplementary Figure 2A), was linearized and introduced into the *C. globosum* NK102 via PEG mediated protoplast transformation. PCR amplification and DNA sequencing revealed that the correct gene deletion occurred at the target locus in transformants Δ*cgtf1*-10 and Δ*cgtf1*-14 (Supplementary Figures 2A,B). Southern blotting and RT-PCR were employed for further verification (Supplementary Figures 2C–F).

### CgTF1 Is Essential for Sporulation

**Figure 2:**
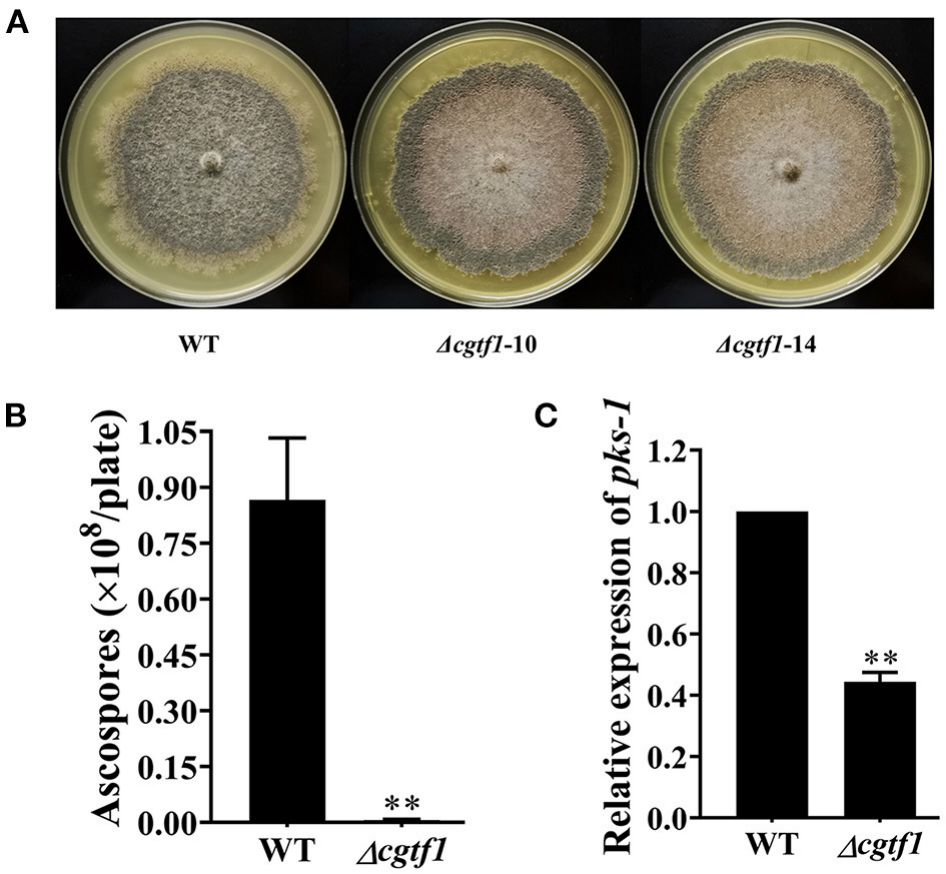
CgTF1 deletion resulted in a severe defect in ascospore development. **(A)** CgTF1 deletion resulted in delayed perithecium formation. The wild-type (WT) was inoculated in PDA medium, and mutants Δ*cgtf1-10* and Δ*cgtf1-14* were inoculated in PDA medium supplemented with 100 mg/L hygromycin B. These plates were unanimously incubated at 28°C for 15 days. **(B)** CgTF1 deletion resulted in a severe defect in sporulation. The indicated strains Δ*cgtf1* and the wild-type were inoculated in the PDA plate and incubated at 28°C for 30 days. **(C)** CgTF1 deletion resulted in downregulation of *pks-1* transcription. Transcript levels of the *pks-1* were detected by qRT-PCR in the WT and Δ*cgtf1* strains. Transcripts of *pks-1* was normalized against *ACTIN*. All experiments were performed in triplicate. There is significantly difference between the WT and CgTF1 null mutants as indicated by two asterisks (*p*-value < 0.01, with *T*-test analysis).

*CgcheR* mutation resulted in the almost complete loss of spores as well as decreased radial growth in *C. globosum* CBS148.51 (Cheng et al., 2021). Consistent with CgCheR, CgTF1 is essential for sporulation. No significant difference was observed in hypha growth rate between CgTF1 null mutants and the wild-type (Supplementary Figure 3). However, CgTF1 deletion resulted in delayed generation of perithecium and inhibition of ascospore development (Figure 2A). No ascospore was observed under the microscope even for a 30 days culture, indicating that the CgTF1 null mutant has lost the ability to produce ascospores (Figure 2B). As *pks-1* was previously proven to be essential for sporulation and pigmentation in *C*. *globosum* NK102 (Hu et al., 2012), we determined the mRNA levels of *pks-1* in *Cgtf1* deficient mutants. As anticipated, *Cgtf1* deletion resulted in down regulation of *pks-1* transcription, e.g., the mRNA level was dramatically reduced to 44% in Δ*cgtf1*-10 (Figure 2C). These results suggested that CgTF1 regulates transcription of *pks-1*.

### CgTF1 Regulates Transcription of *CgcheA* Gene Cluster to Control ChA Biosynthesis

**Figure 3:**
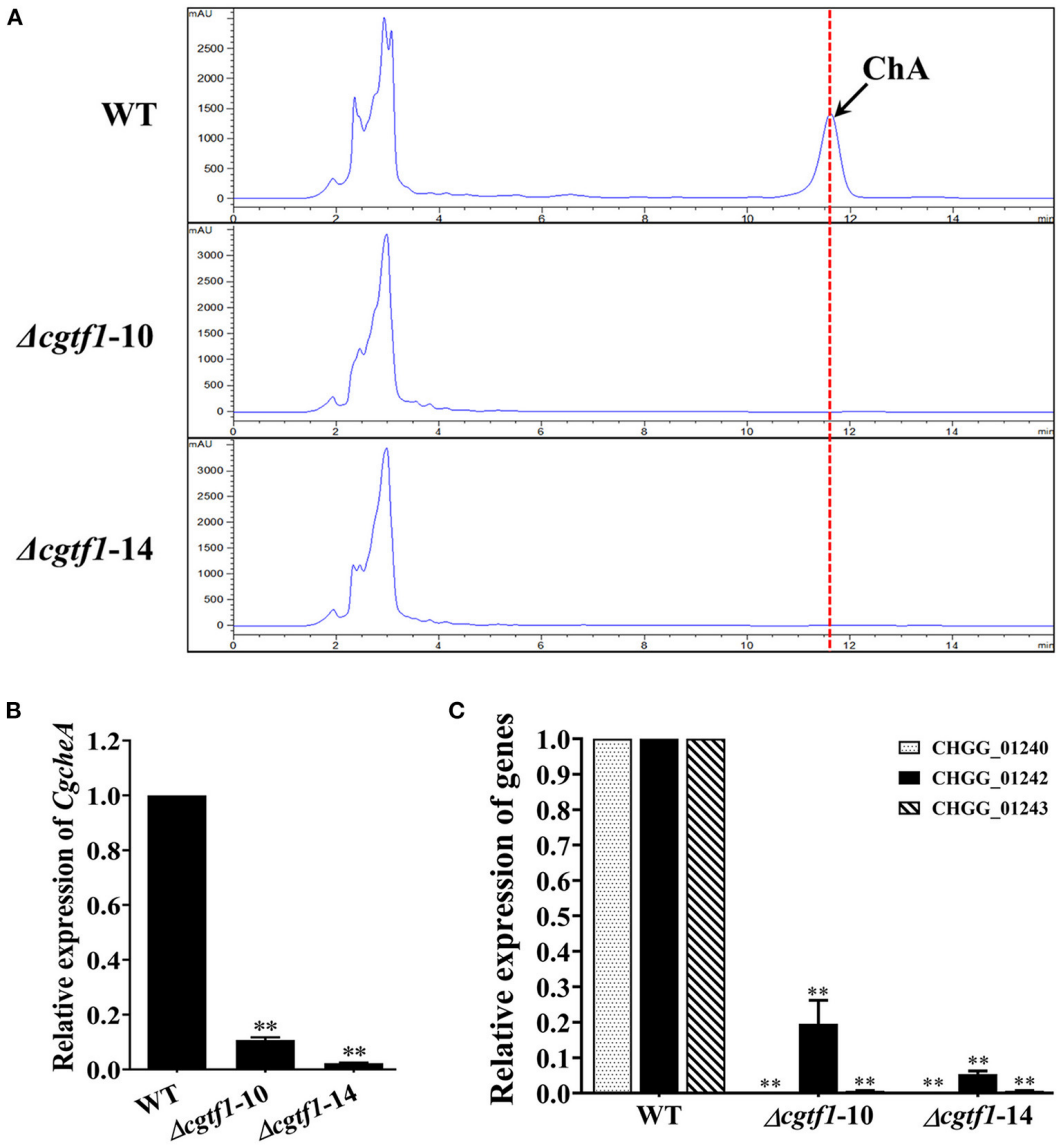
CgTF1 deletion resulted in incapability of ChA biosynthesis. **(A)** The CgTF1 null mutants lost the ability to produce ChA. HPLC analysis on the production of ChA in the wild-type (WT), Δ*cgtf1*-10 and Δ*cgtf1*-14. The dotted line indicates the peak of ChA. **(B)** CgTF1 deletion suppressed transcription of *CgcheA*. **(C)** CgTF1 deletion suppressed transcription of redox enzyme encoding genes in ChA biosynthetic gene cluster. Transcript levels of the CHGG_01240, CHGG_01242-1, CHGG_01243 were detected by qRT-PCR in the wild-type, Δ*cgtf1*-10 and Δ*cgtf1*-14 mutants. All experiments were performed in triplicate. Transcripts of all indicated genes was normalized against *ACTIN*. There is significantly difference between the WT and CgTF1 null mutants as indicated by two asterisks (*p*-value < 0.01, with *T*-test analysis).

CgCheR has been reported to be involved in ChA biosynthesis by activating the transcription of chaetoglobosin biosynthetic genes in a pathway-specific manner (Cheng et al., 2021). To determine the function of CgTF1 in ChA biosynthesis, HPLC analysis was performed to quantify the concentration of ChA in *Cgtf1* deletants. In 7-day-old fermentation broths, no ChA was detected in CgTF1 null mutants (Figure 3A). Subsequently, qRT-PCR was performed to detect the mRNA level of genes within the ChA biosynthetic gene cluster in *Cgtf1* deletants. As shown in Figure 3B, the core gene, *CgcheA* (CHGG_01239) was significantly down regulated in *Cgtf1* deletants. The mRNA level of *CgcheA* in Δ*cgtf1*-10 and Δ*cgtf1*-14 was dramatically reduced to 10.8 and 2.3%, respectively, of that in the wild-type strain. Similarly, CgTF1 deletion also led to a general decrease of transcription of the other genes within the cluster. Most of all, *CgcheB* (CHGG_01240) and *CgcheG* (CHGG_01243) were barely expressed in CgTF1 null mutants (Figure 3C). These results confirmed that CgTF1 regulates transcription of *CgcheA* gene cluster to control ChA biosynthesis as well as CgCheR.

### RNA-Seq Profiling Identifies a Novel Cys_2_His_2_ Transcription Factor CgTF6 Regulated by CgTF1

To assess a global profile of genes regulated by CgTF1, an RNA-Seq profiling analysis was performed to identify the differentially expressed genes associated with the biosynthesis of ChA in the *Cgtf1* deletants Δ*cgtf1*-10 and Δ*cgtf1*-14. Total RNA was extracted from mycelium grown in PDB medium for 4 days for the Illumina HiSeq™ sequencing as described in experimental procedures. The resulting sequences were aligned to the reference genome of *C*. *globosum* CBS 148.51 (assembly ASM14336v1) and the information was used to analyze the DEGs between the wild-type strain and CgTF1 null mutants. DEGs were selected based on the FDR-corrected *p*-value ≤ 0.001 and |Log2 (Ratio value)| ≥ 1. In a total of 1,388 differentially expressed genes, 548 genes were up-regulated, while 840 genes were down-regulated in *Cgtf1* deletants (data in Supplementary Table 2). Consistent with the qRT-PCR results mentioned above, genes within the ChA biosynthetic gene cluster were down-regulated simultaneously in *Cgtf1* deletants (Table 1). However, the gene with the highest gene expression variation (log2 ratio = −4.76) was CHGG_01244, not genes encoding enzymes for synthesis of the skeleton structure of ChA, such as *CgcheA* or *pks-1*. Transcription of genes previously reported to be involved in regulation of ChA biosynthesis was also affected by CgTF1. For example, the expression of *CglaeA* was increased with a gene expression variation of 0.89 in Δ*cgtf1* compared to the wild-type, while the expression of *gna-1* was decreased with a gene expression variation of −0.41 in Δ*cgtf1* compared to the wild-type.

**Table 1:** Expression variation of genes putatively related to ChA biosynthesis detected by RNA-seq profiling.

**Gene symbol**	**Deduced function (homolog)**	**Log2 ratio (WT/TF_KO)**	**FDR**	**Up-or down-regulation(WT/TF_KO)**
CHGG_01239	*CgcheA*, PKS–NRPS hybrid	−0.68	1.26E-102	Down
CHGG_01240	*CgcheB*, enoyl reductase	−1.92	7.97E-10	Down
CHGG_01241	hypothetical protein	−1.42	1.98E-07	Down
CHGG_01242-1	P450	−3.93	5.01E-71	Down
CHGG_01242-2	FAD-dependent oxidoreductase	−3.93	5.01E-71	Down
CHGG_01243	P450 (*gliF*)	−1.2	0.00016	Down
CHGG_01244	hypothetical protein	−4.76	2.47E-51	Down
CHGG_00542	*Pks-1* (*alb1*)	−0.69	0.05	Down
CHGG_01690	*CglaeA*	0.89	1.74E-16	Up
CHGG_10370	*CgveA*	−0.13	7.59E-14	Down
CHGG_09972	*CgsptJ*, histone acetyltransferase	0.16	0.0088	Up
CHGG_03321	*gna-1*, group I Gα protein	−0.41	2.23E-62	Down
CHGG_00688	*pkaR*, the regulatory subunit of the cAMP-dependent PKA	0.95	1.03E-87	Up

**Figure 4:**
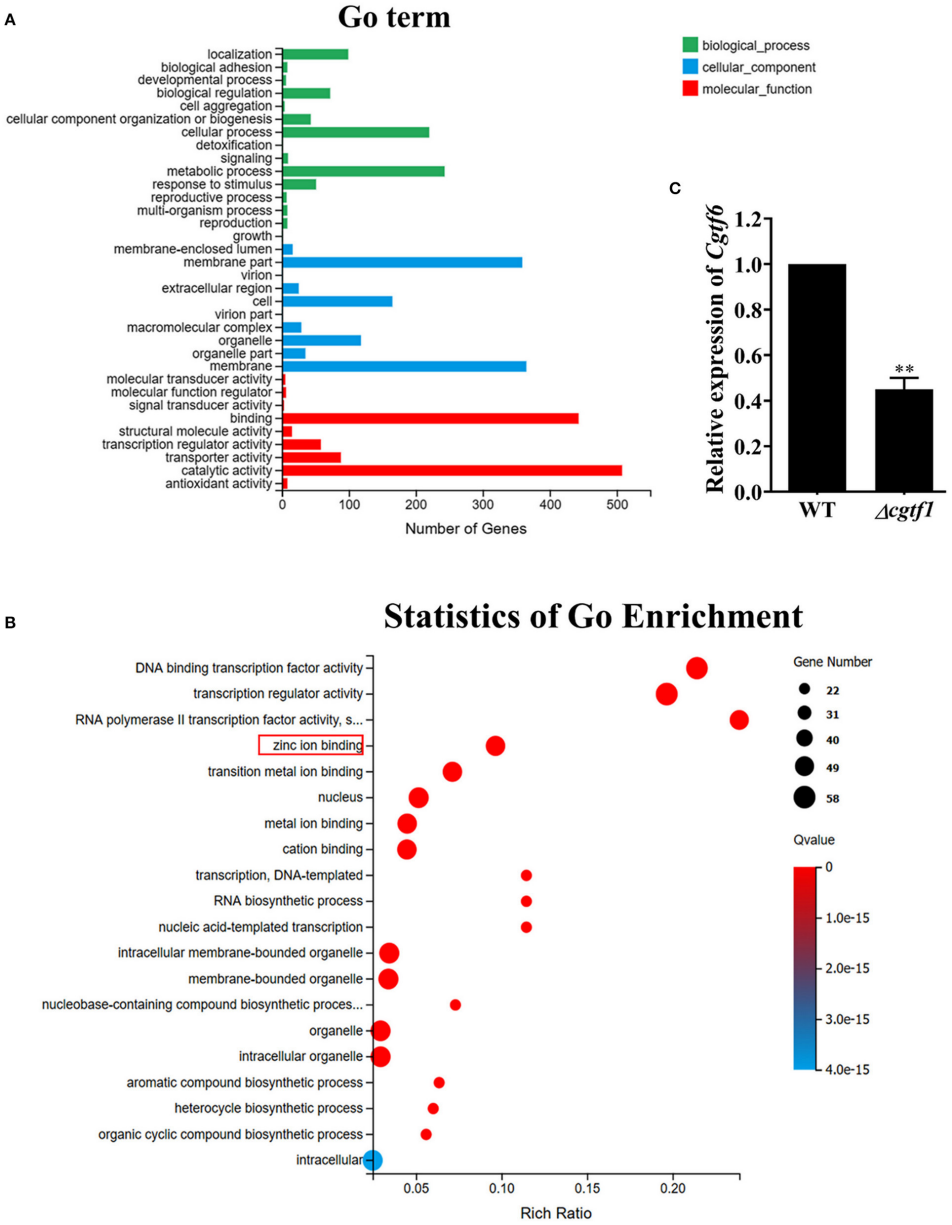
RNA-Seq Profiling identifies a novel Cys_2_His_2_ transcription factor CgTF6 regulated by CgTF1. **(A)** GO annotation of differentially expressed genes between *Cgtf1* null mutant and the wild-type of *C. globosum* strain. **(B)** Statistics of GO enrichment of differentially expressed in GO term of transcription regulatory activity. **(C)** Gene expression of *Cgtf6* in the wild-type (WT) and the Δ*cgtf1*-10. Transcripts of *Cgtf6* was normalized against *GAPDH*. Experiments were performed in triplicate. There is significantly difference between the WT and Δ*cgtf1-10* as indicated by two asterisks (*p*-value < 0.01, with *T*-test analysis).

Gene ontology (GO) analysis for the differentially expressed genes was performed to identify their function. Based on the Blast2GO analysis of sequence homology, 989 annotated sequences that had received Blast hits from the non-redundant NCBI protein database were classified into 28 functional groups under the main categories of the GO classification (Figure 4A and Supplementary Table 2). The top four enriched GO terms in biological process were cellular process (220 genes), biological regulation (72 genes), localization (99 genes), and metabolic process (243 genes). The top four enriched GO terms in cellular component were membrane part (359 genes), cell (165 genes), organelle (118 genes) and membrane (365 genes). Moreover, catalytic activity (508 genes), binding (443 genes), transporter activity (88 genes) and transcription regulatory activity (58 genes) were significantly enriched in the molecular function subgroup. It should be noted that most of the genes in GO term of transcription regulatory activity were putative zinc-binding protein-encoding genes (Figure 4B). It is suggested that the CgTF1 may act as a regional regulator and involve in other secondary metabolism pathways except for ChA biosynthesis. Thus, we screened for genes with high expression variation (|log2 ratio| ≥ 2.00). A total of 335 genes, of which were 7 putative zinc finger transcription factor genes were picked out (Supplementary Table 2 and Table 2). In order to dig the downstream effectors of CgTF1 and reveal the crosstalk between CgTF1 and these zinc finger transcription factors, we selected two genes CHGG_ 02034 (log2 ratio = −3.17) and CHGG_07161 (log2 ratio = −2.68) that with the highest gene expression variation to delete (Table 2).

**Table 2:** Putative zinc finger transcription factor genes with the top expression variation regulated by CgTF1 detected by RNA-seq profiling.

**Gene symbol**	**Deduced function (homolog) or functional domain**	**Log2 ratio(WT/TF_KO)**	**FDR**	**Up-or down-regulation(WT/TF_KO)**
CHGG_02034	GAL4-like Zn(II)_2_Cys_6_	−3.17	1.72E-09	Down
CHGG_07161	ZnF_C_2_H_2_	−2.68	0	Down
CHGG_01338	Fungal_TF_MHR superfamily, GAL4-like Zn(II)_2_Cys_6_	−2.2	2.29E-27	Down
CHGG_07031	Fungal_TF_MHR superfamily, GAL4-like Zn(II)_2_Cys_6_	−2.09	2.20E-107	Down
CHGG_00926	GAL4-like Zn(II)_2_Cys_6_	2.08	1.42E-222	Up
CHGG_02943	Fungal_TF_MHR superfamily	2.05	8.06E-233	Up
CHGG_06050	GAL4-like Zn(II)_2_Cys_6_	2.01	0	Up

We first examined expression levels of CHGG_02034 and CHGG_07161 in the wild type of *C. globosum* NK102. qRT-PCR analysis indicated that CHGG_07161 indeed expressed at a normal level and down regulated in mutant Δ*cgtf1* (Figure 4C), while the expression of CHGG_02034 was repressed in the wild-type (data not shown). Thus, CHGG_07161 was selected for further investigation. CHGG_07161 was located at contig_NT165980.1 of scaffold_5, which was different from *CgcheR* (located at contig_NT165976.1 of Scaffold_1). A single-copy homolog of CHGG_07161 was found in the genome of *C. globosum* NK102, named *Cgtf6*. The 1772-bp gene *Cgtf6* was cloned, subjected to sequencing and submitted to the GenBank database under accession number MT050052. We conducted a Blastp analysis against EggNOG database (http://eggnogdb.embl.de/) for more information about *Cgtf6*. It was indicated that the predicted protein CgTF6 encoded by *Cgtf6* is a 400 amino acids sequence comprises a ZnF_C_2_H_2_ domain (SM00355), which shares a high sequence identity (52.64%) with the gene XP_003662155.1 of *Myceliophthora thermophila* ATCC 42464 (Supplementary Figure 4A).

### Deletion of *Cgtf6* Inhibited Sporulation of *C. globosum*

In order to investigate the function of CgTF6 and figure out the correlation between CgTF1 and CgTF6, we tried to knock out the gene *Cgtf6* by CRISPR-Cas9 system as mentioned above. In order to improve the efficiency of homologous recombination, the distance between two flanking fragments was shortened to 69 bp (Supplementary Figure 4B). The gene deletion construct pCgtf6 was linearized and introduced into the *C. globosum* NK102 via PEG mediated protoplast transformation. Six transformants were identified by diagnostic PCR (Supplementary Figure 4C). Two of them were subsequently subjected to Southern blot analysis. Only Δ*cgtf6*-6 was confirmed the desired disruption of *Cgtf6* by the marker *Hyg*^*R*^ via double crossover at the target site, and with a single-copy of integration (Supplementary Figures 4D,E).

**Figure 5:**
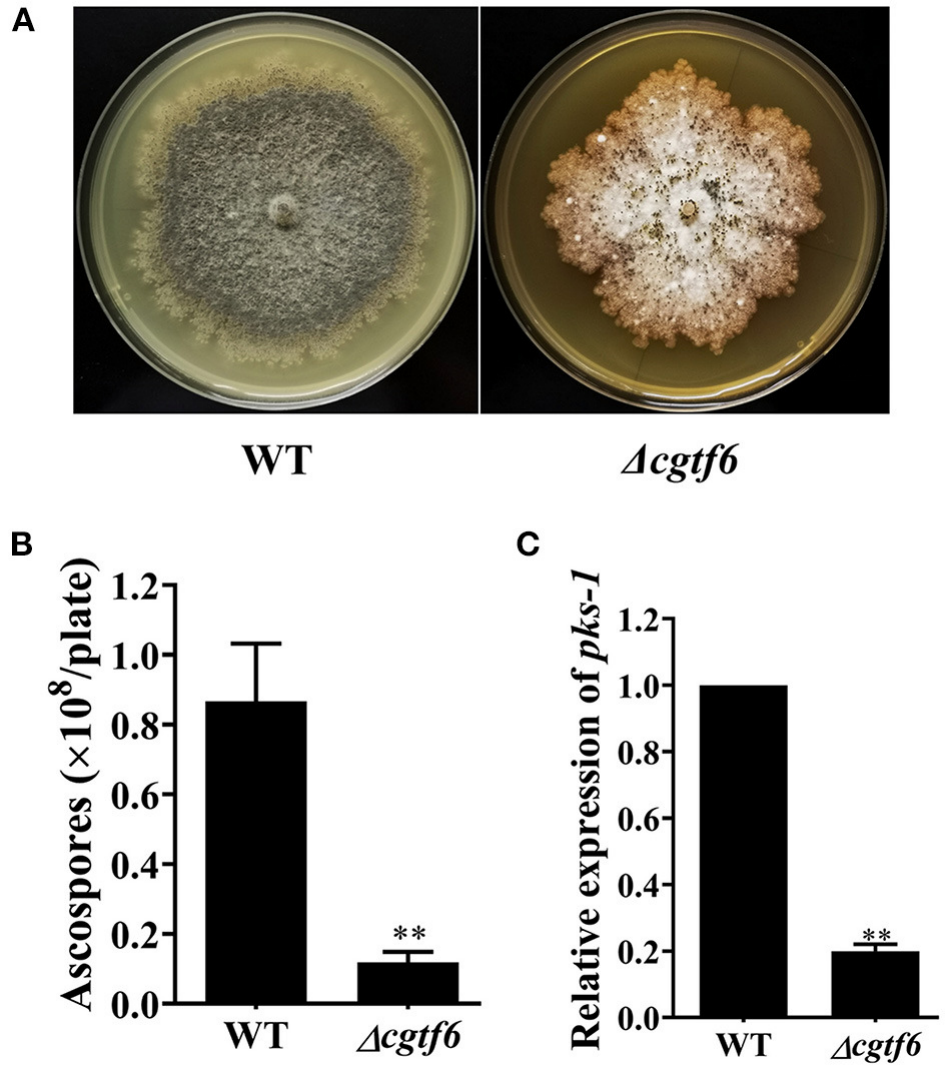
CgTF6 deletion severely inhibited sexual development of *C. globosum*. **(A)** CgTF6 deletion severely inhibited perithecium formation. **(B)** The number of ascospores produced by the wild-type (WT) and Δ*cgtf6*. The indicated strains were inoculated in the PDA plate and incubated at 28°C for 15 days. **(C)** Gene expression of *pks-1* in WT and Δ*cgtf6*. Transcripts of *pks-1* was normalized against *GAPDH*. All experiments were performed in triplicate. There is significantly difference between the WT and Δ*cgtf6* as indicated by two asterisks (*p*-value < 0.01, with *T*-test analysis).

An 8-day comparison on colony diameter as strains growing indicated that CgTF6 deletion has no significant effect on hypha growth rate of *C. globosum* (Supplementary Figure 5). However, CgTF6 deletion significantly inhibited sporulation of *C. globosum*. The number of ascospores produced by the wild type was (8.67 ± 0.16) × 10^7^ per plate, while dramatically reduced to (1.19 ± 0.03) × 10^7^ per plate as CgTF6 deleted (Figures 5A,B). Consistent with this phenotype, the mRNA level of *pks-1* in Δ*cgtf6* was significantly decreased to 19.9% of that in the wild-type strain (Figure 5C).

### CgTF6 Has an Inhibition Effect on ChA Biosynthesis by Repressing Transcription of *Cgtf1*

**Figure 6:**
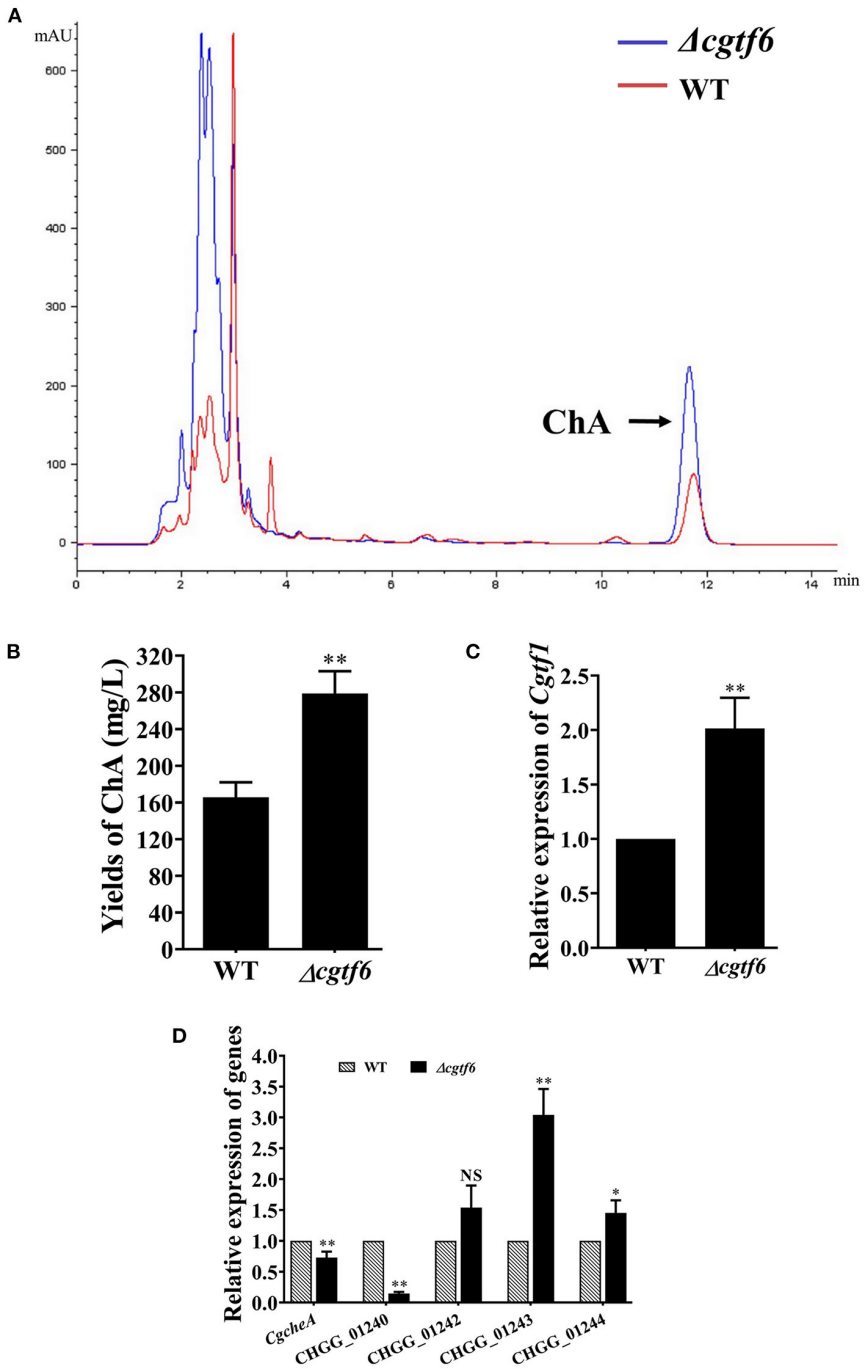
CgTF6 deletion promotes ChA biosynthesis. **(A)** HPLC analysis on the production of ChA in the wild-type (WT) and the Δ*cgtf6*. The arrow indicates the peak of ChA. **(B)** Yields of ChA in WT and Δ*cgtf6* strains. **(C)** Gene expression of *Cgtf1* in the Δ*cgtf6* mutant. Transcripts of *Cgtf1* was normalized against *GAPDH*. **(D)** Gene expression of ChA biosynthetic gene cluster in WT and Δ*cgtf6* strains. Transcripts of all indicated genes were normalized against *GAPDH*. All experiments were performed in triplicate. There is significantly difference between the WT and Δ*cgtf6* as indicated by one or two asterisks (*p*-value < 0.05 = ^*^, *p*-value < 0.01 = ^**^, with *T*-test analysis). NS: no significant difference.

We examined the effect of CgTF6 deletion on ChA biosynthesis. HPLC analysis demonstrated that the yield of ChA in the mutant Δ*cgtf6* was significantly increased to 1.69-fold of that in the wild-type (Figures 6A,B). Considering down regulation of CgTF6 in Δ*cgtf1* mutants (Figure 4C), we speculated CgTF6 acted as an end product feedback effector and interacted with CgTF1 to maintain a tolerable concentration of ChA for cell survival. Thus, deletion of *Cgtf6* probably removed this inhibition effect and promoted ChA biosynthesis. Subsequently qRT-PCR analysis confirmed this speculation. As shown in Figure 6C, transcript level of *Cgtf1* in the Δ*cgtf6* mutant was significantly increased to 2-fold of that in the wild-type. In order to reveal the regulation mechanism of CgTF6 on ChA biosynthesis, we then examine the mRNA levels of other genes within ChA biosynthesis gene cluster in mutant Δ*cgtf6* (Figure 6D). Transcript levels of core genes *CgcheA* and *CgcheB* were down regulated, while two oxidative modification enzymes encoding genes CHGG_01242 and CHGG_01243 demonstrated markedly increase in different degrees. In addition, the mRNA level of unknown gene CHGG_01244 was also increased sharply in Δ*cgtf6*. To sum up, CgTF6 probably inhibited transcription of CgTF1, and then inhibited transcription of modification enzyme encoding genes to suppress ChA biosynthesis.

## Discussion

As a potential biocontrol fungus, *C. globosum* has always been the focus of biocontrol research, but it has not been applied to the field as a commercial product. The low content of active components may be one of the bottlenecks hindering its field experiment. Recently, a growing number of data suggests that the main secondary metabolites of this species, chaetoglobosin A, can be developed in the biocontrol of nematode, sap-sucking pests and phytopathogenic fungi. Unveiling the mechanism for regulation of ChA biosynthesis is the first step toward pesticide development. However, the regulation of fungal secondary metabolism is very complex and operates on different regulatory levels, including pathway-specific and global regulators, signal transduction pathways, and epigenetic control. The role of the global regulator LaeA on ChA biosynthesis has been confirmed by studies of Watanabe and Zou teams (Ishiuchi et al., 2013; Jiang T. et al., 2016). Deletion of either *CglaeA* or *CgveA* led to a significant absence of ChA, whereas overexpression of *CglaeA* resulted in upregulated expression of the ChA biosynthetic gene cluster and significant increased production of ChA, demonstrating positive regulatory activity of *CglaeA* on ChA biosynthesis. Data from Watanabe indicated that biosynthesis of ChA was also affected by a histone acetyltransferase CgSptJ, and deletion of *CgsptJ* resulted in a remarkable reduction in ChA production. Recently, via an established RNAi approach, we have reported that Gα-cAMP/PKA pathway positively regulates pigmentation, ChA biosynthesis and sexual development in *C. globosum* (Hu et al., 2018). In addition, expression of CgSptJ was significantly down regulated in the Gα protein silenced mutant as verified by both RNA-seq analysis and qRT-PCR. With our findings, we hypothesize that CgLaeA, CgVeA and CgSptJ probably work as downstream effectors that dictate expression of the ChA biosynthesis gene cluster by interacting with G protein/cAMP/PKA signaling. However, information is still lacking as to what proteins and transcriptional regulators are responsible for differential expression of the ChA biosynthetic gene cluster either in Gα signaling or in epigenetic modification.

This year, *CgcheR* (CHGG_01237), a predicted Gal4-type Zn_2_Cys_6_ transcription factor gene located in the ChA biosynthetic gene cluster was demonstrated to activate the transcription of ChA biosynthetic genes in a pathway-specific manner (Cheng et al., 2021). In this study, we deleted the homologous gene of *CgcheR* (designated as *Cgtf1*) via the “suicide” CRISPR-Cas9 system in *C. globosum* NK102. Consistent with CgCheR, CgTF1 deletion resulted in significant decrease in the transcription of the ChA biosynthetic gene cluster and consequent reduction of ChA production (Figure 3). CgTF1 deletion also resulted in a severe inhibition effect on sporulation, and the Δ*cgtf1* strain lost the ability to produce ascospores (Figure 2B). Different from CgCheR, CgTF1 deletion has no effect on hypha growth rate (Supplementary Figure 3), and the Δ*cgtf1* strain still can generate perithecium (Figure 2A). Based on the transcriptome analysis of Δ*cgtf1* mutants and the wild-type, we identified the *Cgtf6* (CHGG_07161) gene, which putatively encodes a C_2_H_2_ zinc finger protein. We found that the mRNA level of *Cgtf6* was significantly decreased in Δ*cgtf1* mutants, of which the gene expression variation is highest in zinc-binding proteins encoding genes involved in transcription regulatory activity. Although gene clusters are often coordinately regulated by the cluster-specific transcription factor, some members can be independently regulated. Genes within the ChA biosynthetic gene cluster were independently regulated by CgTF6. Deletion of *Cgtf6* resulted in transcriptional activation and consequent increase in the transcription of the *Cgtf1* gene, CHGG_01242, CHGG_01243 and CHGG_01244, while the transcription levels of *CgcheA* and *CgcheB* were decreased to 73 and 14.8% of that in the wild-type, respectively. Nevertheless, the yield of ChA in the Δ*cgtf6* mutant was increased to ~1.7-fold of that in the wild-type despite the expression reduction of core genes. It was suggested that CgTF6 had an inhibition effect on the transcription of *Cgtf1* and three tailor enzyme-encoding genes.

Furthermore, we found that perithecium and ascospores formed in the Δ*cgtf6* mutant was substantially decreased as compared to the wild-type (Figures 5A,B). Consistent with this observation, expression of *pks-1*, previously reported to be involved in sexual development of *C. globosum*, was dramatically down regulated in Δ*cgtf6* by qRT-PCR analysis (Figure 5C). These data ascertain a positive role of *Cgtf6* in the sexual development in *C*. *globosum*.

Transcriptional regulation is complicated. Transcription factors can bind as monomers, homodimers, heterodimers, or heteromeric complexes, and can work as pathway-specific regulators, self-regulator or broad domain transcription factors. There is considerable evidence that plant and fungi development is synergistic regulated by transcriptional control through hierarchical levels of transcriptional regulatory elements. In *Arabidopsis*, HD-to ZIP IV transcription factor HDG1 and bHLH transcription factors CFLAP1/2 function in a synergistic but ATCFL1-dependent manner to maintain the expression balance of genes related to cuticle development (Li et al., 2016). Plant ALOG (*Arabidopsis* LSH1 and *Oryza* G1) family proteins TTMF, TFAM1 and TFAM2, directly interacted with each other to form heterodimers and function together in regulating reproductive development (Huang et al., 2018). Jamil reported C_2_H_2_-type zinc finger proteins DKZF1 and DKZF2 could synergistically control persimmon fruit deastringency (Jamil et al., 2019). Meng reported transcription factor MrSt12 implicated in the regulation of transcription factor AFTF1 by Fus3-MAPK during cuticle penetration by the entomopathogenic fungus *Metarhizium robertsii* (Meng et al., 2019). Many fungal secondary metabolites production is reported to be regulated both positively and negatively to maintain a tolerable concentration for cell survival. For instance, in addition to the pathway-specific regulations of TRI6 and TRI10, the synthesis of the secondary metabolite trichothecene mycotoxin deoxynivalenil (DON) in *Fusarium graminearum* could be influenced through transcription factors FgSKN7 and FgYAP1. Specifically, FgSKN7 positively regulates DON and FgYAP1 negatively regulates DON (Montibus et al., 2013; Jiang C. et al., 2016).

**Figure 7:**
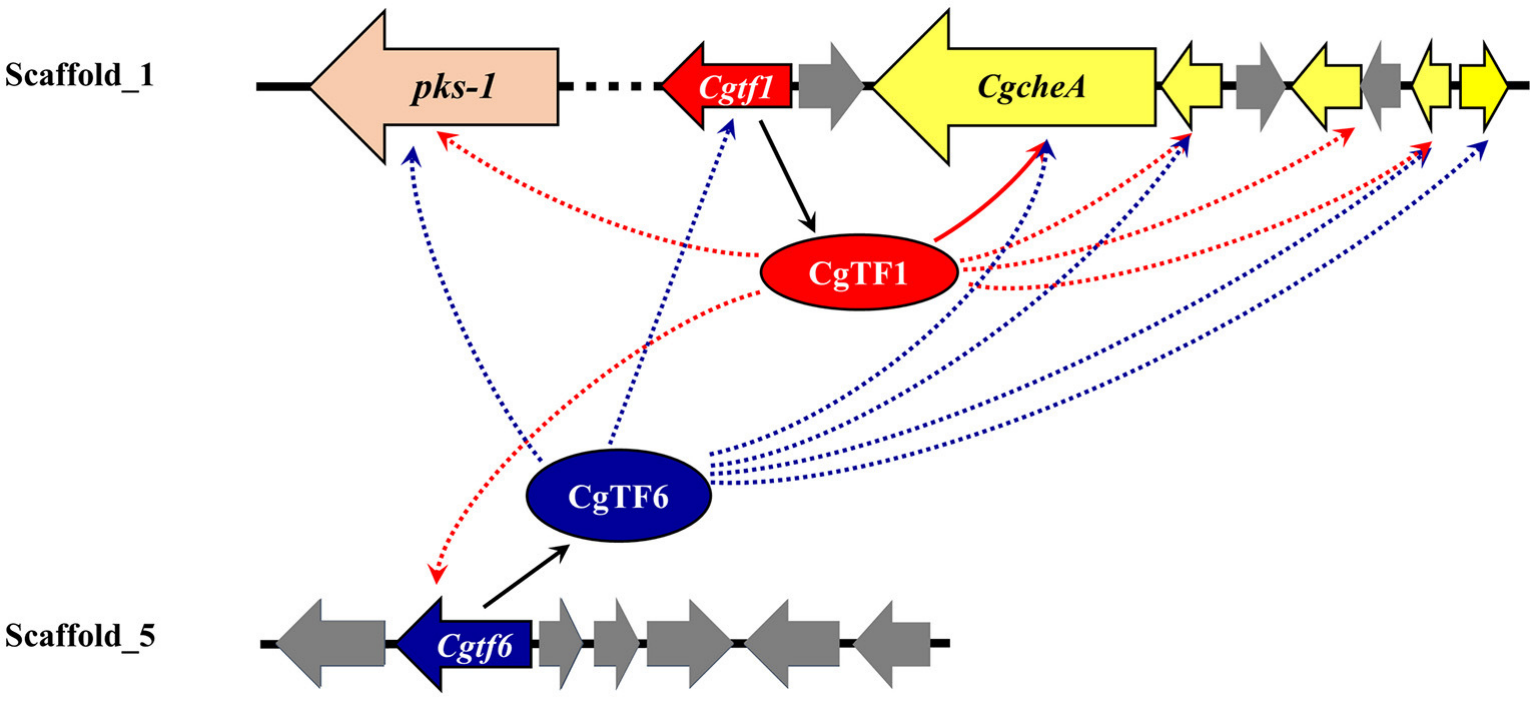
Suggested schematic depiction of the transcriptional regulatory crosstalk between CgTF6 and CgTF1 on ChA biosynthesis in *C. globosum*. Dashed arrows indicate possible activation or suppression by CgTF1 or CgTF6.

In conclusion, these data suggested that CgTF1 acts as an intermediate regulator and positively regulates the ChA biosynthesis. A downstream effector of CgTF1 was a putative C_2_H_2_ transcription factor CgTF6, which had a somehow feedback inhibition effect on ChA biosynthesis. CgTF1 and CgTF6 mutual regulated to maintain a tolerable concentration of ChA for cell survival. Our findings provide additional insight into the regulatory mechanism of the ChA biosynthesis (Figure 7). Future work should be done to uncover downstream pathways regulated by CgTF1 and demonstrate how CgTF1 regulates the downstream effectors for ChA biosynthesis, and ChIP-Seq analysis was considered an efficient way to find downstream effectors of CgTF1. These future studies will shed light on the regulatory pathways for secondary metabolism of *C. globosum* and may provide the reference for further research into other cytochalasans biosynthesis.

## Data Availability Statement

The datasets presented in this study can be found in online repositories. The names of the repository/repositories and accession number(s) can be found in the article/Supplementary Material.

## Author Contributions

XH and XZ conceived and designed the study. YY, BX, QX, YL, and GS performed the experiment. YY, BX, XH, and XZ analyzed the data. YY and XH wrote the original draft. YY, BX, XH, and XZ reviewed and edited the manuscript. XZ funding acquisition, project administration, and supervised the manuscript. All authors approved the final manuscript.

## Funding

This work is supported by the grants from the National Science Foundation of China (#81871629).

## Conflict of Interest

The authors declare that the research was conducted in the absence of any commercial or financial relationships that could be construed as a potential conflict of interest.

## Publisher's Note

All claims expressed in this article are solely those of the authors and do not necessarily represent those of their affiliated organizations, or those of the publisher, the editors and the reviewers. Any product that may be evaluated in this article, or claim that may be made by its manufacturer, is not guaranteed or endorsed by the publisher.
