# Supplementary material for: A Putative C2H2 Transcription Factor CgTF6, Controlled by CgTF1, Negatively Regulates Chaetoglobosin A Biosynthesis in Chaetomium globosum
Source: Front Fungal Biol. 2021 Oct 15;2:756104. doi: 10.3389/ffunb.2021.756104 (PMC10512409; doi:10.3389/ffunb.2021.756104)
Supplement: Supplementary file 1 [file Data_Sheet_1.docx]

Supplementary Material

## SUPPLEMENTARY TABLES

**Supplementary Table 1.** Primers used in the study.

| **Primer name** | **Sequence (5′-3′)** |
| --- | --- |
| N19-F | CCTCGAGTGCCACGCAGATCTGCT |
| N19-R | AAACAGCAGATCTGCGTGGCACT |
| 01237UP-F | GCTTTTTTGTTTTACTCGAGCAGAACTCTACACCGAGAAGGA |
| 01237UP-R | CTGTCGACTCTAGACTCGAGAGGAAGTATGGCAGCAGCAA |
| 01237DOWN-F | GGTAATCCTTCTTTCTCGAGAAGAGGATGGATGGAGATGAAGT |
| 01237DOWN-R | TAGAGGATCCCCGGCTCGAGGTATGCGTAGGTAGGCTATGC |
| 07161-N19-F | CCTCTTACAGCGCGCCGCCCATG |
| 07161-N19-R | AAACGCATGGGCGGCGCGCTGTAA |
| 07161-UP-F | TTTGTTTTACTCGAGGGAGAGTGGAAGATGGACACC |
| 07161-UP-R | GACTCTAGACTCGAGGCGTGGTAGTGAGGAGAAGG |
| 07161-DOWN-F | TCCTTCTTTCTCGAGGGAGTCTATGGAGCGCCTAC |
| 07161-DOWN-R | GATCCCCGGCTCGAGGCGGCCGCGGGCATTCGTAGGGTCTTTCT |
| 01237Probe-F | AGAGGATGGATGGAGATGAAGT |
| 01237Probe-R | GTATGCGTAGGTAGGCTATGC |
| Hyg(s) | ATGAAAAAGCCTGAACTCAC |
| Hyg(as) | GCAAAGTGCCGATAAACAT |
| T01237up-F | CGACAACATTCTGTTCGTGAC |
| THyg-R | CAGGTCTTGCAACGTGACA |
| THyg-F | ACGGCAATTTCGATGATGCA |
| T01237down-R | CACCCACCTCTAGATATGGC |
| L01237Kpn I-F | AAGGATCTGGATCCGGTACCATGGCCTCAGCTGAGGAAAC |
| L01237Hind III-R | CCGCCATATGAAGCTTCCCGAAACTGTTCGTTCGCA |
| Cgtf6-Up-Hyg-F | GCAGCCACCAAAGTGATACAG |
| Cgtf6-Up-Hyg-R | TTCCTTGCGGTCCGAATGG |
| Cgtf6-Hyg-Down-F | GGACCGATGGCTGTGTAGAA |
| Cgtf6-Hyg-Down-R | CCTTGTTGGCGAGGTTGTG |

**Continued to Supplementary Table 1.**

| **Primer name** | **Sequence (5′-3′)** |
| --- | --- |
| q01237-F | GGGAAGGACCGATACCATAAAC |
| q01237-R | TCTAAACCCCATTCTACAACCG |
| qCHGG_01239(s) | CCAGGAGCACGATACCATCTACAC |
| qCHGG_01239(as) | GGCATCCCCATGTAGTTCGTATTG |
| qPKS(s) | ATCTTTCCGCCTAACCCGA |
| qPKS(as) | GTCCTTCGTTTCTGGGTTGTC |
| qCHGG_01240-F | GGTATTACAACGGATGCGACTT |
| qCHGG_01240-R | CGGTAGGAGAACACGCTGAC |
| qActin(s) | AACCGAGGCTCCCATCAAC |
| qActin(as) | TCACGGACGATTTCACGCTC |
| GAPDH-up | CCTGCAAACGATGCCTCAAG |
| GAPDH-down | TATCACCGGTTTTGGCTGCT |
| qCHGG_01242-F | CAGATTAGGGAGCAGTACCCG |
| qCHGG_01242-R | CTCGTCAGCCACCTCTTGTG |
| qCHGG_01243-F | CGGTCTTGCGGCTATTGAT |
| qCHGG_01243-R | GCTGGCGACTTCTTGTCTG |
| qCHGG_01244-F | CACACGCAACGAGTATATCCT |
| qCHGG_01244-R | ATCGTGCTTTGCCGCTTC |
| qCHGG_02034-F | CGGTTATGCGGTGGCTACA |
| qCHGG_02034-R | CTTGACACATGCGACTTGGTT |
| qCHGG_07161-F | CCACAACCACAGTCGTCTC |
| qCHGG_07161-R | TTGTTGCTGCTGCGTCAT |

**Supplementary Table 2.** Summary of all genes that have a difference expression between WT and *△cgtf1* mutants. Data is obtained by RNA-seq profiling analyses. "WT" indicates the wild-type of *Chaetomium globosum* NK102. "TF_KO_1" indicates *△cgtf1-*10 strain. "TF_KO_2" indicates a parallel sample of *△cgtf1-10* strain. "TF_KO_3" indicates *△cgtf1-*14 strain. WT_1, WT_2 and WT_3 indicate three parallel samples of WT.

**SUPPLEMENTARY FIGURES**


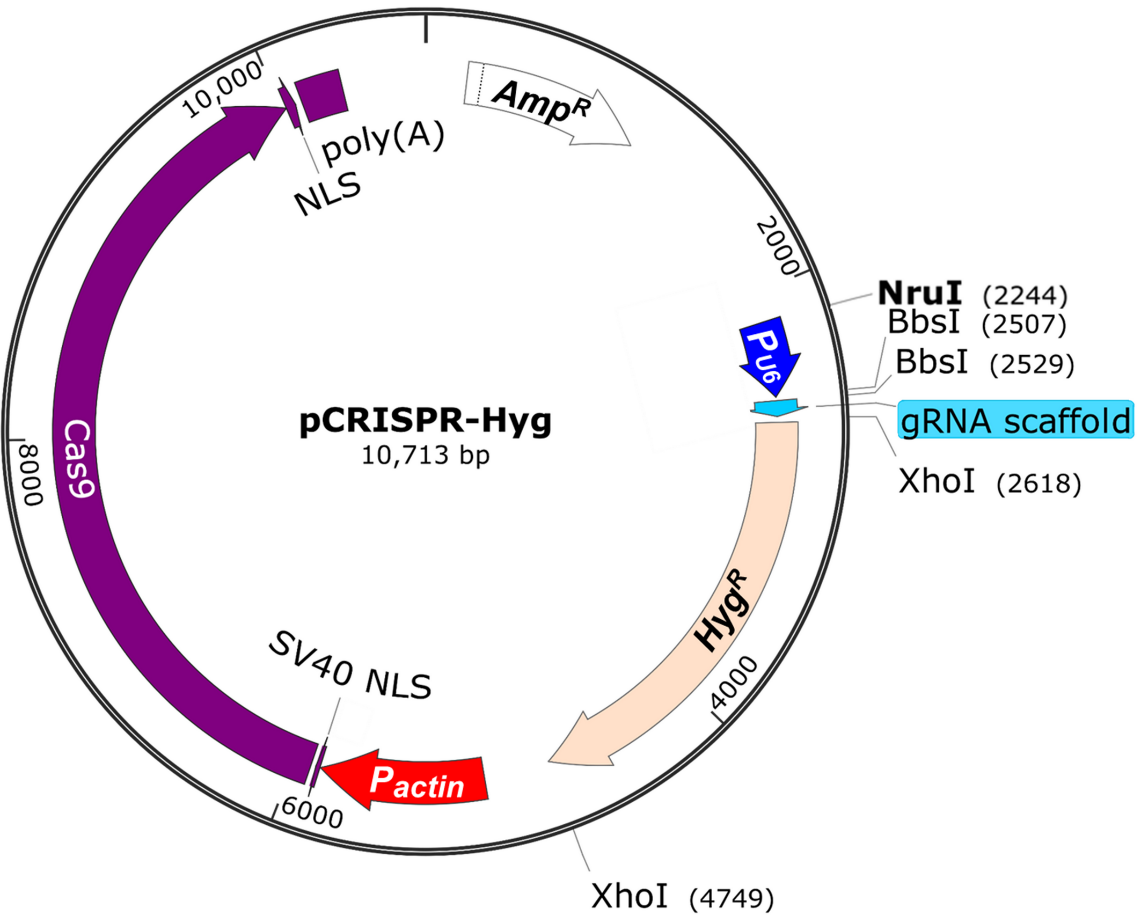


**Supplementary Figure 1.** Diagram of pCRISPR-Hyg. Plasmid pCRISPR-Hyg was used as the vector of the targeted deletion of *Cgtf1* and *Cgtf6*. The promoters of Cas9 and sgRNA were derived from *ACTIN* and *U6* of NK102, respectively. The GN_19_ of targeted loci could be introduced into gDNA scaffold by two adjacent *Bbs* I restriction sites. There is an *Xho* I restriction site for homologous arms insertion at each end of the hygromycin resistance gene. The generated pCgtf1 and pCgtf6 were linearized by *Nru* I.


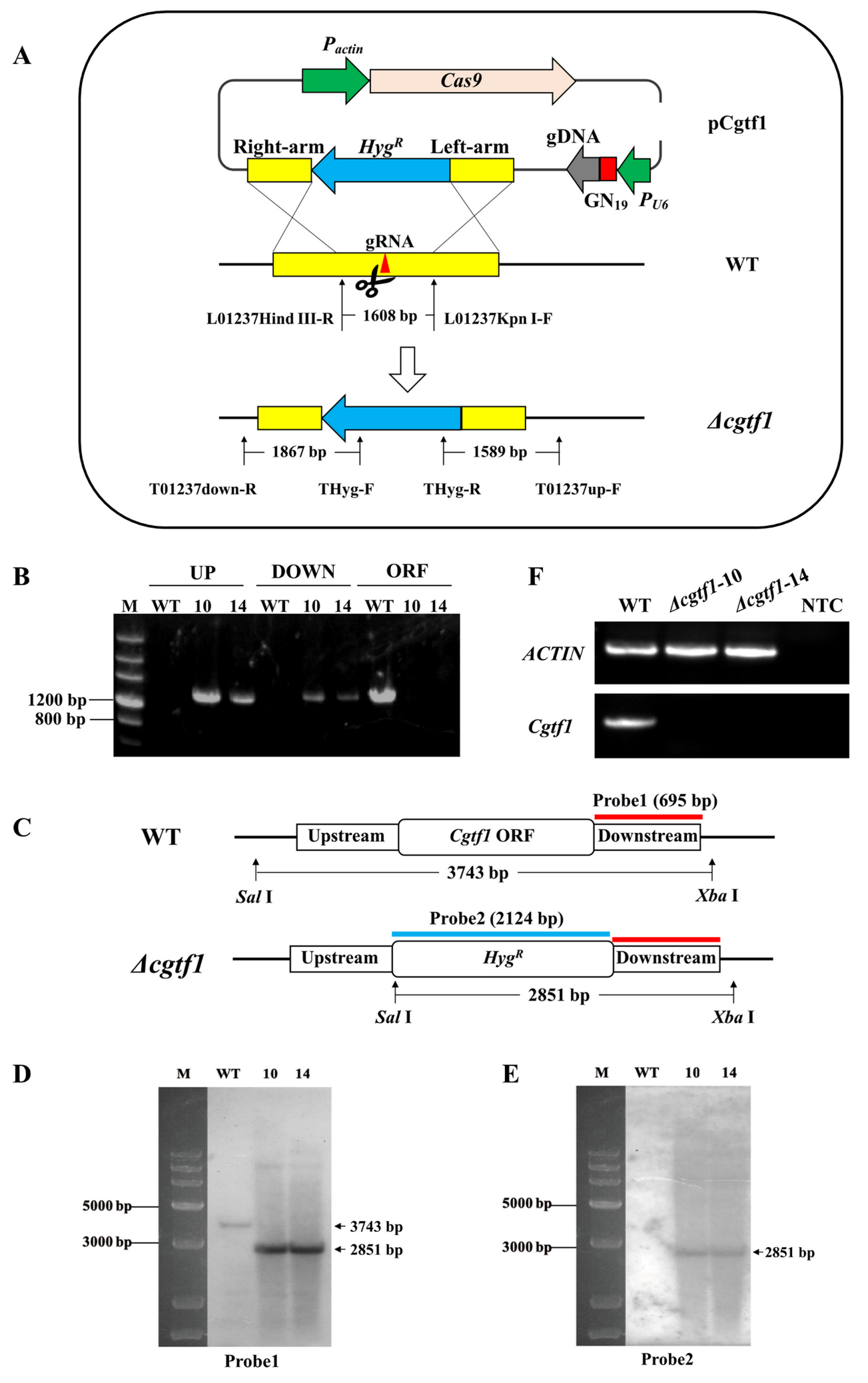


**Supplementary Figure 2.** Targeted deletion of *Cgtf1* via the “suicide” CRISPR-Cas9 system. **(A)** Diagram of the targeted deletion of *Cgtf1* via CRISPR-Cas9-mediated homologous recombination (HR). Cas9 was placed behind an 800 bp promoter sequence of the *ACTIN* from *C. globosum*. The gDNA expression cassette consisting of a targeting sequence, GN_19_, which was fused by overlap PCR to the tracrRNA scaffold under the *U6* promoter of *C. globosum* and included a termination signal of 6 Ts. The gRNA target locus is shown as a red triangle, where a double-strand break was supposed to be generated by Cas9 to promote HR via double cross-over. The primers used for PCR verification are indicated by the arrows. **(B)** PCR screening for *Cgtf1* deletion mutants produced by CRISPR-Cas9-mediated HR. **(C), (D)**, and **(E)** Southern blotting confirmed that *Cgtf1* was deleted as anticipated. Two randomly selected mutants, *Δcgtf1*-10, *Δcgtf1*-14 and the wild-type (WT) were analyzed. The membrane was probed with a 695 bp fragment of *Cgtf1* downstream that was amplified by PCR with the primers 01237 Probe-F/01237 Probe-R. Genomic DNA was digested with *Sal* Ⅰ and *Xba* Ⅰ. The WT had a wild-type *Cgtf1* band (3743 bp), and the mutant strains were supposed to have a shorter band (2851 bp). In a second blot, DNA were probed with *Hyg^R^* cassette from pUCATPH. M: *Trans* 15K DNA Marker (TransGen, Beijing, China). **(F)** Detection of *Cgtf1* mRNA by reverse transcription PCR for further confirmation. *ACTIN* mRNA was used as the internal control. Gels were run under the same experimental conditions. NTC: no template control.


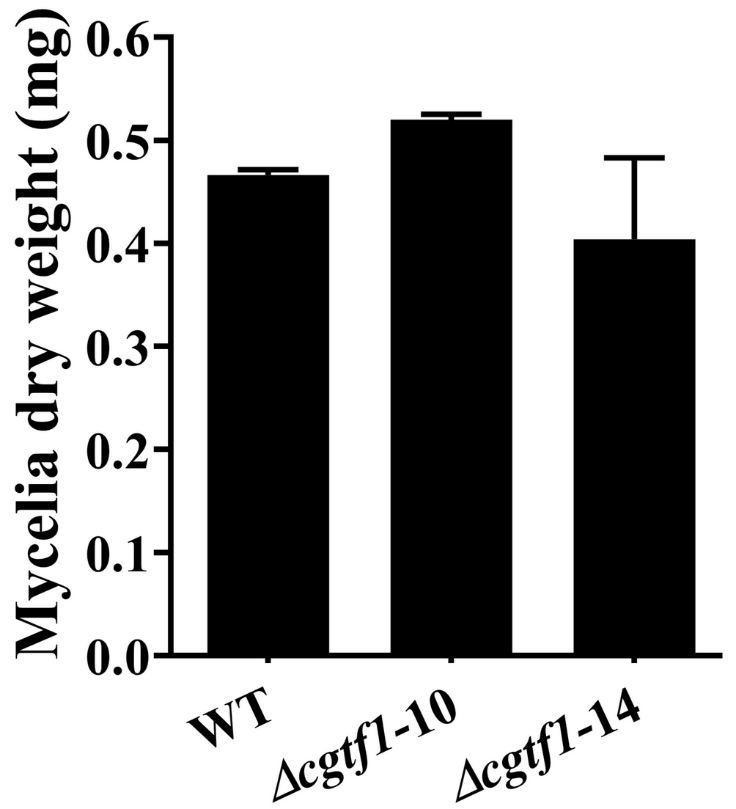


**Supplementary Figure 3.** Deletion of *Cgtf1* has no significant effect on mycelium growth rate. Mutants *Δcgtf1-*10, *Δcgtf1-*14 and the wild-type (WT) strain were inoculated in 100 mL PDB medium and incubated at 28℃ for 7 days with shaking at 200 rpm, then the mycelium was separated from the broth by the Büchner funnel and subjected for freeze drying.

**(A)**


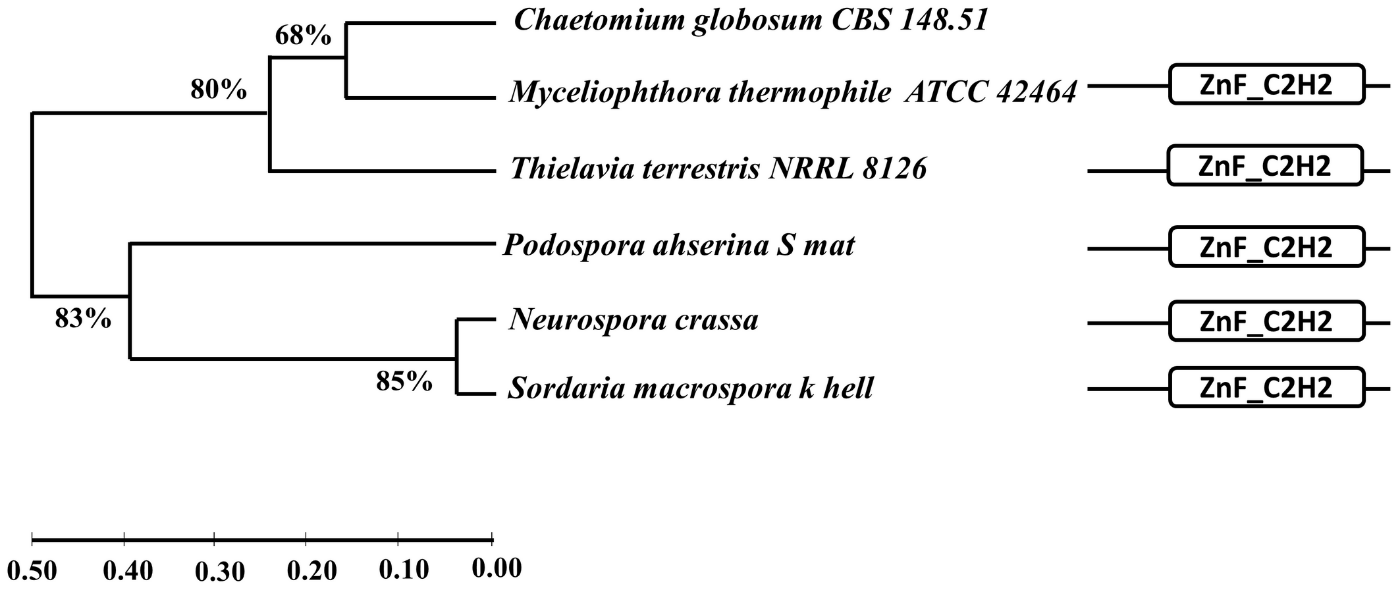


**(B)**


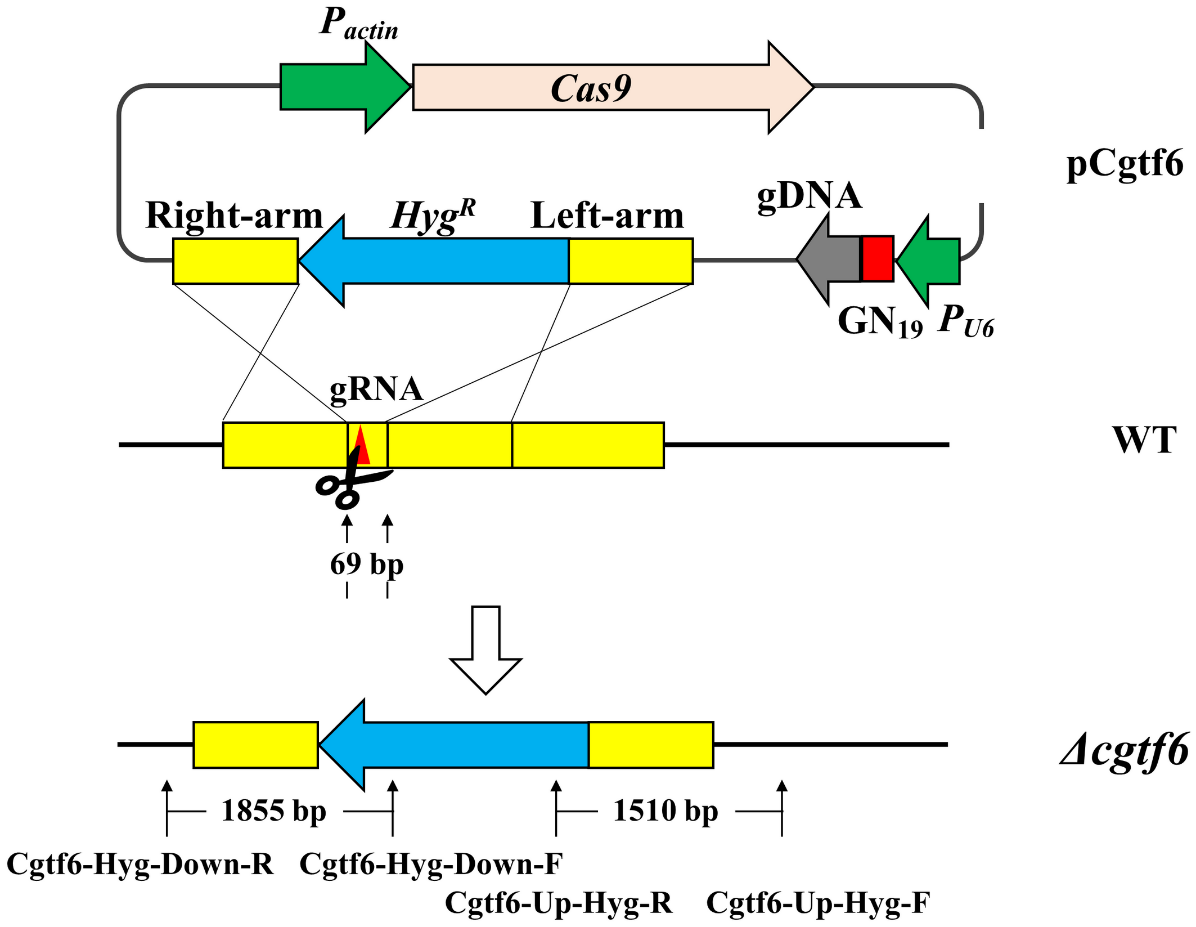


**(C)**


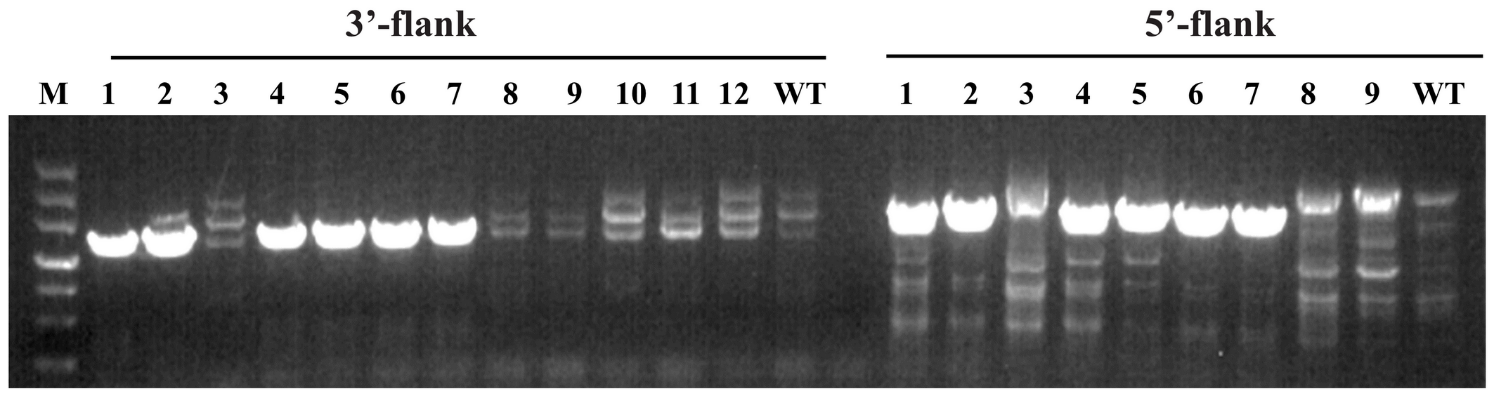


**(D)**


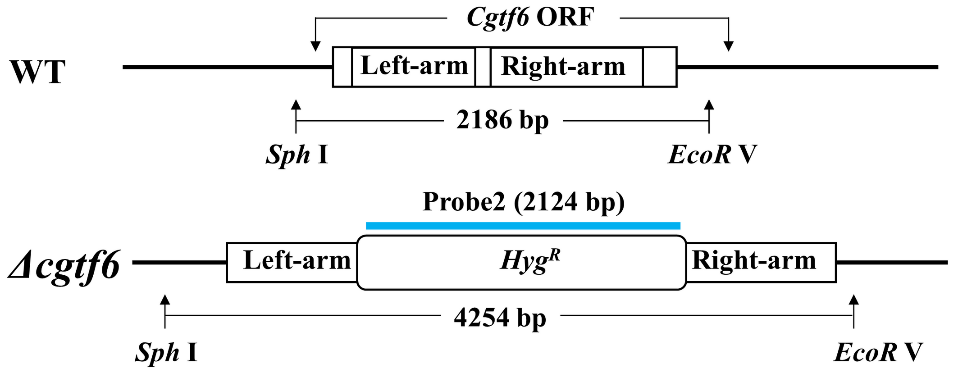


**(E)**


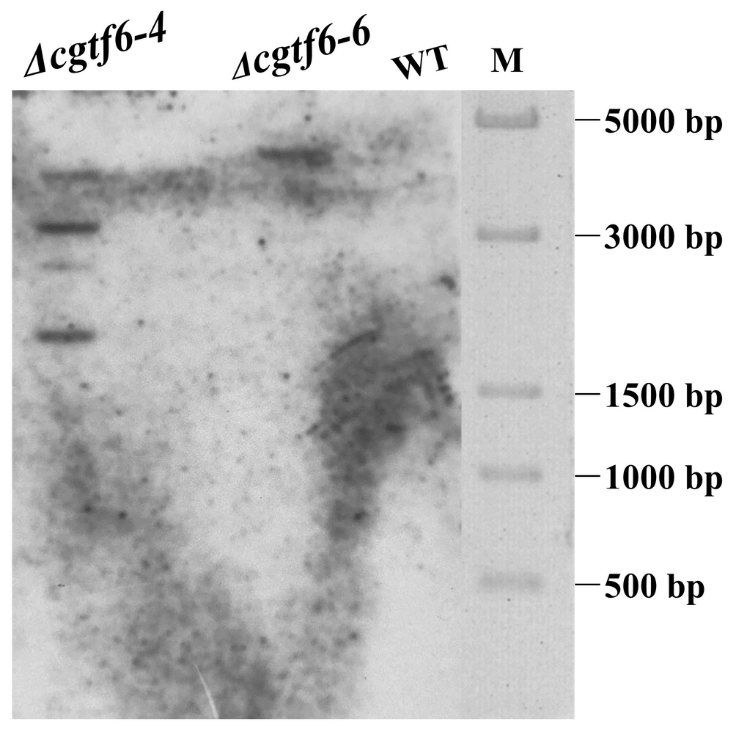


**Supplementary Figure 4.** Targeted deletion of *Cgtf6* via the “suicide” CRISPR-Cas9 system. **(A)** Structure analysis and function prediction of CgTF6 in EggNOG database. **(B)** Diagram of the targeted deletion of *Cgtf6* via CRISPR-Cas9-mediated HR. The gRNA target locus is shown as a red triangle, where a double-strand break was supposed to be generated by Cas9 to promote HR via double cross-over. The primers used for PCR verification are indicated by the arrows. **(C)** PCR screening for *Cgtf6* deletion mutants produced by CRISPR-Cas9-mediated HR. M represents Marker Ⅲ (TIANGEN, Beijing, China). **(D)** and **(E)** Southern blotting to confirm that *Cgtf6* was disrupted as anticipated. Two randomly selected mutants, *Δcgtf6*-4, *Δcgtf6*-6 and the wild-type (WT) strain were analyzed. The membrane was probed with a 2124 bp *Hyg^R^* cassette from pUCATPH. M represents *Trans* 15K DNA Marker (TransGen, Beijing, China).

**
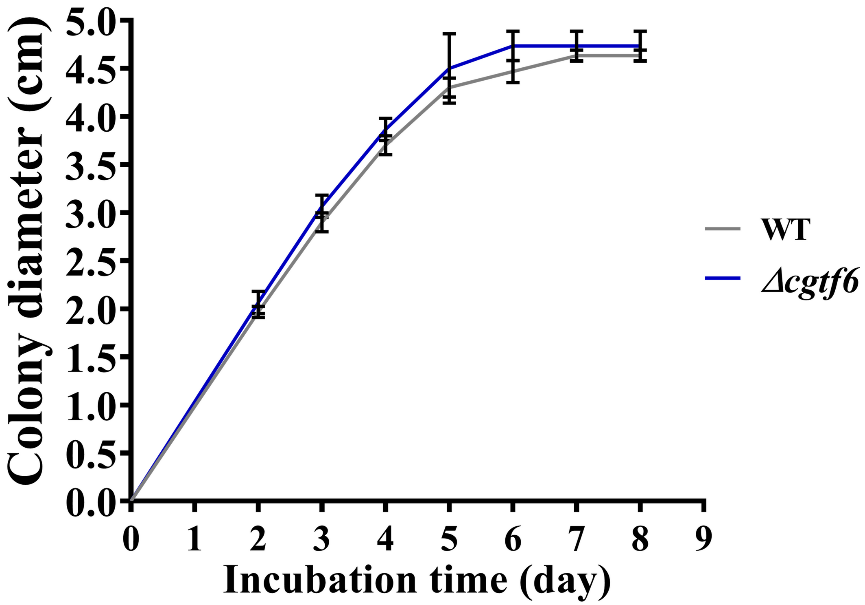
**

**Supplementary Figure 5.** Colony growth curves of the wild-type (WT) and the *Δcgtf6* strains from day 0 until 8 days. All indicated strains were inoculated in PDA plates and incubated at 28℃. There is no significantly difference between the mutant and WT.
